# Standards of suitability for the management of chronic obstructive respiratory diseases

**DOI:** 10.1186/2049-6958-9-65

**Published:** 2014-12-18

**Authors:** Claudio M Sanguinetti, Nicolino Ambrosino, Filippo Andò, Fernando De Benedetto, Claudio F Donner, Stefano Nardini, Mario Polverino, Roberto Torchio, Guido Vagheggini, Alberto Visconti, on behalf of the Consensus Conference 2013 Group

**Affiliations:** Managing Director Multidisciplinary Respiratory Disease, Senior Consultant Respiratory Diseases, Quisisana Clinical Center, Rome, Italy, Rome, Italy; Center of Respiratory Weaning and Rehabilitation Auxilium Vitae, Volterra, (PI) Italy; Pneumology Unit, G.Martino General Hospital, Messina, Italy; Pneumology Unit, SS.Annunziata General Hospital, Chieti, Italy; Mondo Medico, Multidisciplinary and Rehabilitation Outpatient Clinic, Borgomanero, (NO) Italy; Pneumology Unit, General Hospital, Vittorio Veneto, (TV) Italy; High Specialty Provincial Pulmonologic Unit, “Scarlato” Hospital, Scafati, (SA) Italy; Lung Function and Sleep Unit, AOU S. Luigi, Orbassano, (TO) Italy; Internal Medicine and Respiratory Diseases, Center of Respiratory Weaning and Rehabilitation Auxilium Vitae, Volterra, (PI) Italy; Scientific Secretariat, AIMAR, Arona, (NO) Italy

**Keywords:** Acute and chronic therapy, Acute exacerbations, COPD, Diagnosis, Guidelines, Management, Smoking, Spirometry, Standards of suitability

## Abstract

**Background:**

Chronic Obstructive Pulmonary Disease (COPD) ranks third as cause of mortality and disability-adjusted life years (DALY) worldwide and also in Italy it imposes a huge health, social and economic load. Early symptoms of COPD are often disregarded by patients and physicians, spirometry is underutilized, and the diagnosis is delayed till the disease has reached a distinct severity level. Despite the availability of various guidelines, the behavior of health workers involved in the management of COPD is still rather unlike. These considerations are the reason why in October 2013 AIMAR (Interdisciplinary Scientific Association for Research in Lung Disease) devised and organized a “Third Consensus Conference”, aimed at pointing out the standards of suitability for COPD management. In this context three important topics of discussion were identified: early and more widespread diagnosis, management of acute and subacute phases, long-term assistance to chronic patients.

**Methods:**

The procedure recommended by the Italian Health Superior Institute (ISS) for Consensus Conferences organization was applied. The Conference was structured in three sessions, each dealing with one of the above mentioned topics and including a short update of the subject-matter and presentation, discussion and voting of some statements with a choice ranging from total agreement to total disagreement or no knowledge. The results of voting were eventually recorded in the document, reviewed by an independent jury, that forms the substance of this paper.

**Results:**

The essential role of spirometry, the need for distinguish between different COPD phenotypes, and the obligatoriness to base on the blood gas analysis findings the long-term oxygen therapy, were largely agreed, as well as the need for interventions aimed at decreasing the rate of acute exacerbations. More specific topics like the use of noninvasive ventilation, recognizing the factors affecting outcome and mortality, the choice of pharmacological and non pharmacological treatments in COPD patients led to lively discussing, but they did not always reach the total agreement, probably because of insufficient familiarity with these problems and of diversities in organization and instruments availability. The chronic respiratory assistance was treated with particular regard to smoking cessation, whose implementation is still insufficient. Many doubts rose due to uncertainty, lack of ability and standardization of procedures, insufficient institutional support, and difficulties to realize a network for assistance to chronic patients.

**Conclusions:**

The results of this Third Consensus Conference revealed some certainties and many doubts and diversities of view also on topics whose importance is well demonstrated in scientific literature. Thus, there is still a long distance to cover before reaching a suitable standardization of COPD management and such situation urges the need for improving not only the health professional’s operativeness but also the organizational support by competent institutions. In this context some initiatives organized by AIMAR in cooperation with other respiratory scientific societies and patients’ associations are going on.

**Electronic supplementary material:**

The online version of this article (doi:10.1186/2049-6958-9-65) contains supplementary material, which is available to authorized users.

## Background

Disability and mortality due to noncommunicable diseases are still a relevant worldwide problem, and a 25% reduction of the mortality due to these diseases is the target established by 2025 by United Nations (UN) and World Health Organisation (WHO) in individuals aged 30–70 years [1–3]. Among noncommunicable diseases Chronic Obstructive Pulmonary Disease (COPD) ranks third as cause of mortality and disability-adjusted life years (DALY) [4]. Also in Italy respiratory diseases are the third cause of mortality [5] and COPD, presumed affecting 5% or more of adult population, imposes a considerable health, social, and economic burden [6–9], all the more that the few reported data likely underestimate the real prevalence of the disease, thus leading to its undertreatment [10]. In fact, COPD is often diagnosed at an advanced severity stage, when acute exacerbations, emergency unit accesses, and the need of pharmacologic and non-pharmacologic costly treatments frequently occur. Early COPD symptoms are often overlooked by patients or their physicians because considered an unavoidable consequence of smoking instead as important signs of an incipient disease that more or less rapidly will become irreversible, progressive, and severely disabling [11–16]. Also spirometry, a critical examination for COPD diagnosis and/or confirmation is underutilized [17], and not rarely the diagnosis is established on clinical grounds only [18]. Despite the availability of several international and national COPD guidelines [6, 7, 19], the behavior of medical and non-medical health workers in the management of chronic respiratory diseases (CRD) is still very dissimilar. It is thus mandatory to improve the suitability of diagnostic and therapeutic interventions, that should be effective, safe, and efficient in order to decrease the burden imposed by these diseases. On the other hand, also the organization of the National Health Service (NHS) should be updated and adapted to the needs of CRD, since the assistance is still mainly based on hospital ground, while WHO recommends a patient-centered continuity of assistance [20]. In this context is really mandatory that all persons who at whatever level are involved in the management of CRD be scientifically competent and professionally trained to observe well-defined organizational arrangements in order to conduct at best their tasks. Such an exigency refers not only to the patient’s care, but also to an efficient organization of institutional and administrative functions. In this respect, the regional compartmentation of NHS makes difficult, if not impossible, to uniform the management of CRD, also because useful pilot experiences experimented in some Italian regions are not always followed in other regions as would be hoped for.

Based on the above considerations, AIMAR (Interdisciplinary Scientific Association for Research in Lung Disease), after organizing two similar conferences in 2007 [21] and 2010 [22], in 2013 devised and organized the Third Consensus Conference (CC) in Respiratory Medicine with a new format aimed at involving all health framework engaged in the management of CRD, from the general practitioner to district and hospital specialists, administrative directors, together with experts of health organization and management, besides patient’s associations representatives. In the context of suitability of chronic obstructive disease (mainly COPD) management, three issues requesting greater attention were identified: the problem of a more widespread and earlier diagnosis, the correct treatment of acute and subacute phases of the disease, the adequate assistance and monitoring of chronic patients. All these topics were discussed in particular about needs and priorities, actors, and competences.

## Methods

The CC followed the procedure recommended by the National Health Institute (ISS) for CC organization [23]. The Promoting Committee, *i.e.* the AIMAR Executive Committee, appointed a technical-scientific committee to identify the topics to be discussed in the three session as above mentioned. Each session included a president, responsible for the organization of the session, and a streamleader charged with an updated revision of the topic to be discussed and with the preparation, together with an experts group, of few statements to be discussed in each plenary session. In each session a group of discussants from different contexts (hospital and district specialists, general practitioners, patient’s associations representatives etc.) promoted the discussion of the statements, and an independent jury similarly composed by health professionals from various contexts, commented on the results emerged from the different sessions of the conferences and reviewed the document derived from these results. With this structure more than one hundred persons have been involved in the CC (Additional file 1). In each session the streamleader first updated the audience on the topic to be discussed, then he presented one at a time the statements prepared for that session. Each statement was then put to voting and each participant gave his/her vote by an electronic system choosing one of the different opinions reported in Table 1.

**Table 1:** **Graduation of opinions expressed on each statement by participants**

1)	Totally agree
2)	Very much agree
3)	Partially disagree
4)	Totally disagree
5)	Don’t know

The statement was considered approved if the percentage of participants who voted “totally agree”, or the sum of percentages of those who voted “total agree” and “very much agree” was greater than 80%. Otherwise, the statement was further analyzed and discussed and then voted again: it was considered approved if the above conditions were reached, otherwise it remained not approved.

## Results and discussion

### First session “The problem of diagnosis”

**Figure 1:**
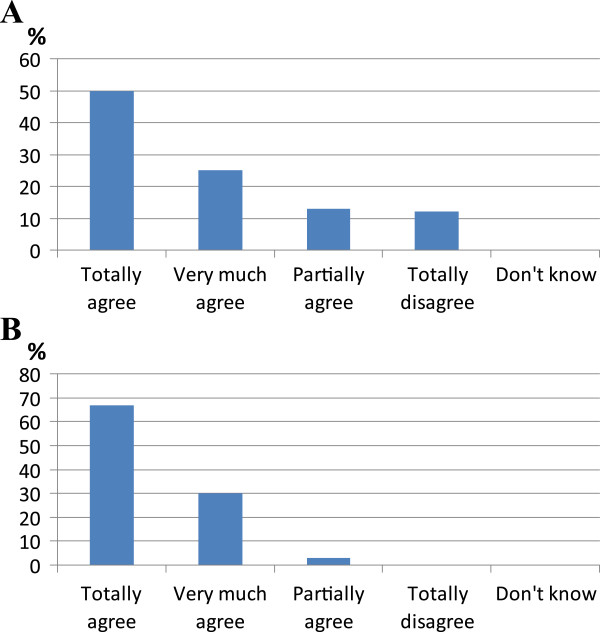
**Results of voting of statement about the need of spirometry for COPD diagnosis. A**: results of first voting; **B**: results of second voting.

In a first statement it was affirmed that COPD diagnosis cannot be performed without a spirometry test in order to verify the presence and quantify the degree of bronchial obstruction. A first voting did not yielded sufficient agreement (Figure 1A), and the consequent discussion revealed a too long waiting time for the examination to be done and the cost of patient’s participation to the spirometric test as negative factors impairing a more extensive use of spirometry. A second voting reached the approval of the statement (Figure 1B). During the discussion the fact that some Italian regions established a target of spirometry implementation in at least 90% of patients also emerged.

Most international guidelines and more recently the document drawn up by Italian Scientific Respiratory Societies together with an Association of Italian General Practitioners, the National Agency for Health Services and the Italian Health Ministry, all agree that COPD diagnosis must be based on respiratory symptoms, risk factors exposure, and demonstration of airflow obstruction with spirometry, together with other lung function tests when necessary [7, 19, 24].

The diagnostic characterization of COPD patients, aimed to distinguish between prevalence of emphysema and chronic bronchitis, has been agreed as desirable by the majority of participants (86%). In fact, in recent past these different disease entities have been grouped under the same definition of COPD because they often coexist in the same patients, especially when they are smokers, but they have different physiologic consequences, induce a different decline of respiratory function, and could likely take advantage of different therapeutic approaches [25, 26]. Based on lung function (spirometry, lung mechanics and diffusion) and radiographic investigations it is generally possible to reveal the presence of either pathological entity and – to some extent – quantify the relative importance when they coexist [27]. The clinical value of diagnosing emphysema resides in that lung hyperinflation, dyspnea and exertion intolerance are predictors of poor survival independent of level of airways obstruction. Besides, the severe hyperinflation often affects diastolic function of left ventricle and cardiac output especially during exercise [28].

Looking at the compelling need of achieving a more diffuse and earlier COPD diagnosis, another statement pointed out that all smokers complaining of respiratory symptoms should be addressed to a spirometry test and this assertion was immediately accepted by 94% of voting people. This is in accordance with the diffuse view that diagnosis should be based on a screening of symptomatic subjects at risk rather than of general population, where the screening would present a reduced cost/benefit ratio because COPD prevalence seems to be low in general population and instead very high in smokers aged over 40 years [29]. Such an approach is endorsed by WHO in the GARD (Global Alliance for Chronic Respiratory Disorders) document, where it is suggested that each subject at risk should perform a spirometry test [30]. General practitioner (GP) is charged with the active search of new COPD patients by using suitable questionnaires [31–33] allowing to find people potentially affected with this disease. Long lasting cough and expectoration, relapsing and hardly remitting infectious episodes of airways, and above all the dyspnea out of proportion in relation to effort or to individuals of the same age, when present, should be interpreted by GP as a need for further investigation with spirometry and/or specialist’s consultation. In this respect the recent Italian document on COPD management [7] recommends performing a simple spirometry test (flow-volume curve) to all subjects at risk and a global spirometry to those with respiratory symptoms.

In order to further clarify the diagnosis in terms of clinical and functional approach, another statement presented to discussion and voting supports that in subjects with decreased lung volumes without obstruction other investigations should be done to exclude restrictive diseases like the neuromuscular ones, and it was approved by more than 80% of participants. Restrictive diseases are caused by decreased lung or chest wall compliance, weakness of respiratory muscles, loss or collapse of lung parenchyma, or by a combination of all the above alterations. According to ERS-ATS guidelines [24] a restrictive defect must be diagnosed only by the reduction of total lung capacity (TLC) because vital capacity (VC) can be decreased by the parallel increase in residual volume (RV). After excluding pulmonary or chest wall alterations, a neuromuscular disease should be suspected and rapidly diagnosed because in some cases (myasthenia, multiple or disseminated sclerosis) useful treatments can be adopted to support an incipient respiratory failure [34–36].

The last statement voted in this session was relative to the need of a blood gas analysis to diagnose respiratory failure to be treated with oxygen therapy and it was agreed by the great majority of the attending people (90%). Italian guidelines for long-term oxygen therapy [7, 37], recommend this treatment in patients with documented respiratory failure who present an arterial oxygen pressure (PaO_2_) steadily ≤ 55 mmHg or borderline hypoxemia (PaO_2_ 56–60 mmHg) in presence of stable polycythemia, pulmonary hypertension, tissue hypoxemia, ischemic cardiomyopathy. Guidelines on diagnosis and treatment of stable COPD, published in 2011 by American College of Physicians, American College of Chest Physicians, American Thoracic Society, and European Respiratory Society [38], recommend oxygen therapy also with Pulsoxymetric saturation (SpO_2_) < 88% based on the opinion that pulse oximetry substantially superseded blood gas analysis in outpatients. However, it is well known that oximetry may yield inaccurate or erroneous results in case of hemodynamic instability, presence of carboxyhemoglobinemia, anemia, jaundice, and cutaneous pigmentation [39]. Even more important, oximetry does not give any information about carbon dioxide blood levels, and in case of hypoxemic-hypercapnic respiratory failure oxygen therapy without ventilator support may aggravate the hypercapnia [40]. This is the reason why the statement refers to Italian guidelines.

### Second session “Management of acute/subacute stages”

The deleterious effects of acute exacerbations of COPD (AECOPDs) on respiratory function, clinical symptoms and outcome were first taken into account. In fact AECOPDs, especially when they lead to hospital admission, negatively affect the course of COPD through respiratory function deterioration, onset of cardiovascular complications, skeletal muscles weakening, worsening of quality of life, increased risk of relapses, hospital re-admissions and mortality [41–43]. In this respect great importance have some measures like promoting patient’s adherence to treatment, adequate drugs prescription, programs of respiratory rehabilitation [44–46]. Thus, the first statement of this session concerned the need for interventions aimed at decreasing the rate and severity of AECOPDs as above indicated and it was agreed by all voting persons (100%).

Some patients, because affected with AECOPD particularly severe or because they need particular treatments not feasible at home or specialistic treatments like noninvasive ventilation (NIV), have to be hospitalized [47, 48]. In this context, particularly important is the evaluation of whether and when to address the patient to hospital admission. For this assessment, some principles have been identified that represent the matter of a second statement: the decision to hospitalize one patient should be based on the severity of symptoms, presence of comorbidities, and degree of patient’s self-sufficiency at home. Pulse oximetry is suitable to evaluate an exacerbated patient at home or at primary care level, whereas the evaluation of an exacerbated patient at admission to hospital should always include blood gas analysis, electrocardiogram, chest x-ray, laboratory examination (hemochrome plus cytologic formula, electrolytes, theophylline level, blood culture in case of fever). This statement was accepted by 85% of participants.

The mortality of hospitalized patients may be increased by some factors such as the development of respiratory acidosis, the severity of dyspnea, and comorbidities. Moreover, frequent hospitalizations for AECOPD are associated with a decreased survival in the mid and long-term [49–53]. These considerations formed the subject of the third statement and the content of this statement was agreed by the great majority of participants, while 10% of them affirmed they did not know these problems.

**Figure 2:**
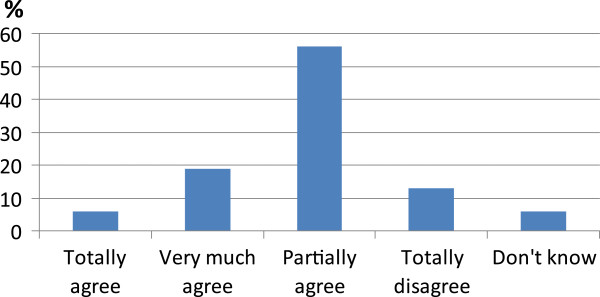
**Results of voting of statement about pharmacologic treatment of AECOPDs.**

As to pharmacologic treatment of AECOPDs, another statement declared that: short-acting beta-2 agonist bronchodilators (SABA) are generally preferred; further studies are needed to verify the effectiveness of long-acting beta-2 agonist (LABA) or antimuscarinic (LAMA) bronchodilators, associated or not with inhaled corticosteroids (ICS); systemic corticosteroids induce a clinical improvement, also when administered for less than 14 days [54, 55]; antibiotics are at present indicated in presence of signs of bacterial infection (increased volume and purulence of sputum), even if the positive influence on outcome would be more evident in severe exacerbations [56, 57]. This statement raised a wide and lively discussion, nevertheless the majority of attending people said they only partially agreed (Figure 2), likely because this statement deals with different pharmacological approaches, that should be discussed singularly. Anyway they are based on strong evidences in the literature also confirmed in clinical practice.

Concerning the non pharmacologic treatment of AECOPDs, a further statement affirmed that: oxygen should be administered to all hypoxemic subjects with a target SpO_2_ 88-92%; high flow oxygen therapy is associated with a worse prognosis [58, 59]; NIV should be recommended in AECOPDs with ventilatory failure, because it can improve the outcome of severe exacerbations [60, 61], as demonstrated by its more diffuse use in last decades [62–66]; respiratory rehabilitation is a safe and effective treatment immediately after the acute episode, able to improve the outcome and decrease re-admissions [45, 46]. The statement was not approved, probably for the same reasons expressed for the precedent statement: in fact, while 76% of voting people agreed, 18% admitted they did not know. Thus, a substantial agreement about oxygen therapy and NIV was not reached according to the pre-arranged criteria, but the participants saying they did not know these problems, and likely also part of those who only partially agreed, definitely influenced the negative results of voting.

Finally, the last statement of this session concerned the assistance out of hospital and was based on the following assertions: the greater part of costs relative to AECOPDs is determined by hospital admission and correlated with the clinical severity, with a large geographical variability, and linked to treatment protocols [67, 68]; early discharge from hospital may be favored by the presence of effective services of assistance at home. Selected patients should be admitted to intermediate institutional health structures intermediate between hospital and home, even if the advantages of these structures in terms of treatment efficacy, patients preference and costs still have to be defined [69–71]. The prevention and prompt treatment of AECOPDs should be the main objective of primary care and an active intervention should include the reduction of risk factors for AECOPDs (smoking cessation, vaccination), inclusion in programs of respiratory rehabilitation, individualized strategies for the management of long-term control therapies [72–74]. All the principles suggested in this last statement were accepted by the majority of voters, who recognized the importance of preventing AECOPDs, especially at general practice level, and reducing costs due to hospital admissions through a shorter hospital stay and a more constant and effective home assistance.

### THIRD SESSION “Organizing the assistance to chronic patients”

This session dealt with the present standards relative to chronic respiratory patient’s care to define both operational aspects of long-term assistance and smoking cessation, an intervention defined fundamental in scientific literature and nevertheless widely disregarded in clinical practice. In patients affected with COPD, smoking cessation may slow down both disability and death, and the patient who continues to smoke can be considered strongly smoke-addict. Thus, smoking cessation is thought a critical therapeutic measure and this treatment must include intensive intervention with pharmacologic and psycho-behavioral therapy. Based on the data from literature and guidelines, a first statement affirmed that active smoking in respiratory patients is just a disease to be treated with drugs and periodic assistance. In a first voting this statement did not reach approval (75% agreed, 23% disagreed). In the subsequent discussion some criticisms emerged that impair a correct and comprehensive assistance to smoker COPD patient: lack of well defined national standards and specific training of physicians and health workers; lack of time to devote to smoking management; no refundability of treatments. However, the opinion of participants at the second voting did not substantially change (78% agreed, 21% disagreed). In recent years smoking cessation has been found not only to slow down the disease progression towards more severe stages and disability, but also to decrease mortality through its effect on smoke-related comorbidities [6, 75]. The minimal advice afforded by physicians may result insufficient in strongly nicotine-dependent smokers and a more intensive treatment seems mandatory because a dose–response correlation between intensity of intervention and its efficacy has been demonstrated [76]. Unfortunately, in Italy medical-assisted smoking cessation is not included in basic principles of care, is not taught in universities, is a voluntary-based practice, and the costs of treatment are at patient’s expense.

**Figure 3:**
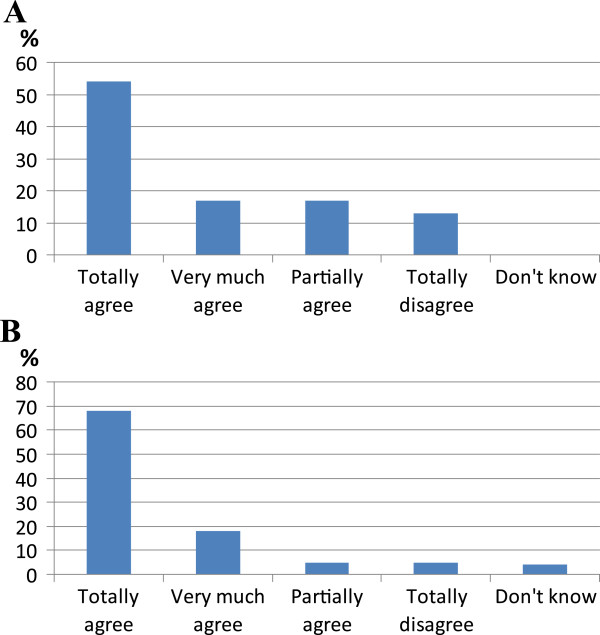
**Results of voting of statement about responsibility of respiratory specialist for organizing a network of assistance to COPD patients. A**: results of first voting; **B**: results of second voting.

To improve the patient-centered assistance to those affected with COPD, a chronic disease with periods of exacerbation and others of clinical stability, is strictly necessary that hospital and territory form a network to guarantee the continuity of care and successfully treat frequent comorbidities in chronic patients. There is no clear definition of this network and of the precise responsibilities yet, and in a second statement it was enounced that the responsible of forming a network for health assistance of COPD patients is the respiratory specialist. This statement was not accepted in this way at first voting (Figure 3A) and a lively discussion took place that evidenced a wide difference between Italian regions or local places where there is a respiratory specialist for outpatients and those where there is not. Several attending physicians affirmed their difficulty to form a similar network owing to their incompetence, to lack of operational instruments and relative budget. The statement was thus modified as follows: the respiratory specialist is the coordinator of assistance activities for COPD patients, and with this formulation it was approved (Figure 3B). It should be noted that also in this case the criticisms are mainly attributable to NHS, because it still does not supply economic, logistic and instrumental resources (also in terms of communication and information technology) to improve the interrelationships among different specialists. Also the deficiency of medical and nursing staff in pneumology units is at the root of the poor enthusiasm to become responsible of a network without human resources to do it.

To accomplish the continuity of care it is mandatory that all who are involved share standardized interventions based on the best evidences of institutional guidelines. In this context it has proposed the statement that operational references should mainly be the institutional national documents [6, 7]. This statement was not approved (46% agreed, 55% disagreed) likely because – as emerged from discussion – the best known guidelines are those published by GOLD [19] and only a lower part of voting people knew the national documents, even if some of them pointed out that it is preferable, if not mandatory, to refer to institutional national documents because they are a guide not only for health workers but also for administrators. A second voting did not reach the approval again even if it substantially approached the agreement (79% agreed, 16% disagreed). Further discussion, with a clarification of the advantages inherent to the use of guidelines adapted to national reality, led to a satisfactory acceptance of the statement (83% agreed, 10% disagreed). GOLD guidelines diffusion started more than one decade ago and since not long ago they were the only ones known in Italy, thus it is not surprising that the greatest majority of physicians refer to them. This is confirmed by another multicentric survey among general practitioners [77] where 62% declared they refer to GOLD guidelines and only 16% to institutional national guidelines, while 22% do not use guidelines at all. In the last two years, however, both specialists and general practitioners began to use institutional guidelines more and more, also because these guidelines have been drawn up through a collaboration of different medical and non-medical components involved in respiratory assistance. During the discussion many respiratory specialists also expressed various criticism about the adequacy/inadequacy of GOLD guidelines to define the suitability of COPD management, starting from the definition of COPD itself, a common umbrella comprising very different clinical entities where recently the concept of phenotype has been introduced. Thus, if on one hand there is the GOLD container from which lastly goes out a unique therapeutic solution, on the other hand there is the need for separating different diseases with unlike therapeutic approaches and outcomes. This induced in last years GOLD committee to add complex diagrams, not rarely open to criticism, in an attempt to depict a more comprehensive image of the COPD patient previously restricted by the sole functional classification. In this respect the discussion emphasized the innovative and more adherent to real life management proposed by the institutional national guidelines.

A controlled self-management may be very useful in a chronic disease and the training of COPD patients for this purpose seems really valuable. Another statement just dealt with this important issue affirming that the main reference to learn the self-management of COPD is the Pneumology Unit and this statement was almost unanimously accepted (91%). In fact, all the participants to the conference recognized that the professional contribution of all health workers in Pneumology Units is the most qualified reference for patient’s education so as Diabetology Units are for diabetic patients.

Integrated care and long-term monitoring out of hospital are essential needs of a chronic disease like COPD. Thus, it was presented for discussion the statement that to accomplish these needs teleassistance is not indispensable, but at first it was not agreed (73% agreed, 24% disagreed). The discussion on this issue evidenced that the negative form with which the statement had been presented could have influenced the voting. Anyway, a part from this consideration, the pessimistic result derives from an honest realism based on the daily difficulties generally encountered in recording and consulting even banal clinical information, because most clinical data frequently are hand-written, and this situation is shared also by administrative structures. This also implies that obtaining and studying epidemiological COPD data is really difficult. A recent Italian study about the main chronic diseases, comparing the prevalence data coming from the administrative database with a sample from general practitioners and with the ISTAT (Italian Institute for Statistics) estimates, found a good correlation among the different sources relatively to diabetes, heart failure and ischemic cardiomyopathy, while there was an underestimation of administrative data compared to those from general practitioners [78] for respiratory diseases, possibly due to a lower availability of management tools in this field. However, the proposal of this statement was useful to assess the different importance attributed to ICT (Information and Communication Technology) by respiratory specialists compared to other professional operators, to point out again the incapacity of NHS to timely supply ICT, the conspicuous differences regarding ICT endowment in various Italian regions or even in different districts of the same region, and the lack of ICT instruments also in many hospitals. After all these issues were discussed, the following voting approved the statement (84% agreed).

Finally, the participation of Pneumology Unit personnel to the establishment and organization of intermediate structures between hospital and territory for post-acute COPD patients was dealt with in the last statement of this session. This statement was not agreed (73% agreed, 26% disagreed) and during the subsequent discussion some perplexities rose relative to the almost absolute lack of intermediate structures in our country, as well as to chronic lack of medical and nursing personnel. In fact, many voting participants pointed out that it is impossible to discuss about programming and organizing the assistance to post-acute patients, who sometimes request also complex treatments, without logistic and human resources. These reservations emerged so strong and evident that, nevertheless the role of respiratory specialist in this contest was clear to everybody, the percentage of those who disagreed with the statement was even increased at the second voting.

The last voting clearly demonstrated that the improvement of care quality, while being a main task of health operators, cannot outlook the administrative support, that is the NHS. A study on the suitability of COPD management performed in an Italian region already demonstrated that the effort to improvement afforded by clinical health personnel is not sufficient without the resources supported by the NHS [79]. In a recent document [80] a respiratory working group evidenced the responsibilities for COPD management: the State and NHS are responsible for programming (by allocating the relative funds) the most suitable preventive actions (some shared by all chronic diseases) and integrating health services. Otherwise, neither the good will of professionals can be sufficient.

## Conclusions

The Third Consensus Conference in Respiratory Medicine dealt with “technical” problems concerning COPD management also from a present and future organizational point of view. Where guidelines are unequivocal or sufficiently shared, or alternatively different guidelines propose the same message, we did not find significant uncertainties. Thus, it is not surprising that an easy agreement has been reached about classical “issues”, so as that spirometry is mandatory to make COPD diagnosis (while this opinion is not always put into practice). Less expected seems that diagnosis should first lead to definition of emphysema or chronic bronchitis prevalence, and spirometry findings have to be further investigated with a more complete functional examination together with imaging data acquisition if necessary. In fact, the prognostic implications and the possible diversity of therapeutic approaches in the two COPD phenotypes, besides the possibility that different diseases from those causing obstructive defects may be present, make necessary that the specialists clarify the most complex situations. The problem would instead be relative to the technological, staff and budget resources necessary to perform in-depth investigations or early diagnosis in all symptomatic smokers, whose appropriateness/obligatoriness has been enthusiastically agreed.

The document proposed by the Italian respiratory societies together with a scientific society of general practitioners with an institutional endorsement [7] proposes simple spirometry to screen smokers and global spirometry for symptomatic individual at risk. To put into practice these recommendations, scientific societies should promote local trials, supported by the Health Ministry and Regional Government, aimed at verifying feasibility, effectiveness, and efficiency of different modalities of screening with spirometry. In fact spirometry may be done in different settings: by GP or his nurse in his surgery [81]; in the GP’s surgery by technicians made available by the Pneumology Unit; by trained personnel in a pharmacy [82]; in a structure of the Social and Health District.

There is a firm conviction that long-term oxygen therapy should be based on repeated blood gas analyses. In this context the task of scientific societies would be to organize audit to verify the adherence not only of physicians both to guidelines and to regional laws when present, but also of patients to prescribed O_2_-therapy. In addition, it seems appropriate that guidelines would include the screening of active smoking in subjects prescribed long-term oxygen therapy to intensively assist them to give up smoking.

The second session of this conference resulted particularly interesting because, differently from the first session, it dealt with problems faced with other health professionals besides the respiratory specialist. In fact, the management of COPD patients, in addition to assistance to smoking cessation, includes the monitoring of disease outcome in order to adapt the treatment to the real severity stage, and the education to a correct use of inhalers, all accomplished by a working group of which respiratory specialist is part [83].

In this session a moderate amount of “don’t know” was recorded, that means a certain level of uncertainty about the proposed criteria for hospitalization. The same occurred as to factors that affect prognosis during and after hospitalization of patients with AECOPDs. Probably, the acknowledgment not to know the problems was mainly belonging to non pneumologists, so as the opinion about pharmacologic and non-pharmacologic treatments, that is issues of close specialist competence. The last statement of the second session, dealing with the assistance out of hospital, while agreed by the majority of participants, showed however some perplexities deriving from the different regional situations in terms of services supply.

The third session recorded the lower percentages of agreement because some problem rose, as the doubts about the reference documents and guidelines, the difficulties to combine scientific evidences with organizational and clinical issues, uncertainty on own role into the organization, uncertainty/fragmentation of the organizational context, lack of objective data.

AIMAR, in cooperation with other institutions and scientific societies, is going to realize several initiatives aimed at resolving doubts and uncertainties about who does what and also at improving the collection of data. Among these initiatives, three are particularly worthy, also because they represent an answer to criticism emerged in this CC.

The first one concerns a pneumologic network to standardize the activities to be done on smokers with respiratory morbidity and consists in: a) drawing up a document of health policy summarizing the position and responsibilities of respiratory specialists towards smoking habit; b) implementation of an intervention protocol on smokers in the context of respiratory medicine (according to “asthma-like” model of ACCP); c) establishment of a network of pneumology units active on smokers in accordance with the health policy document (point a) and with the intervention protocol (point b) above mentioned; d) accreditation of pneumology units of the network with the endorsement of ISS Smoking, Alcohol and Drug Observatory (OSSFAD) through processes of internal and external audit by AIMAR and other scientific societies.

The second initiative is organized in cooperation with a large-sized association of elderly people to define excellence requirements for pneumologists, GPs, pharmacists, patients and their care givers in the management of chronic respiratory diseases.

The third initiative together with Health Federation and Municipalities Association consists in preparing a schedule to measure the suitability of COPD management.

The results of these initiatives and others in progress will form the matter of discussion in the “Fourth Consensus Conference in Respiratory Medicine”.

## Electronic supplementary material

Additional file 1: **Participants in Consensus Conference and relative function.** (XLS 117 KB)
